# Comparison of the Diagnostic Efficiency of Mediastinal Lymph Node Endobronchial and Endoesophageal Ultrasound with Transcervical Extended Mediastinal Lymphadenectomy in Operable Non-Small Cell Lung Cancer

**DOI:** 10.3390/cancers17132207

**Published:** 2025-07-01

**Authors:** Michal Wilkojc, Pawel Gwozdz, Sylweriusz Kosinski, Juliusz Pankowski, Artur Szlubowski, Aleksandra Kiszka-Wilkojc, Wojciech Czajkowski, Robert Kwiatkowski, Marcin Zielinski

**Affiliations:** 1 Department of Thoracic Surgery, Pulmonary Hospital, 34-500 Zakopane, Polandmarcinz@mp.pl (M.Z.); 2 Department of Anesthesiology and Intensive Care, Pulmonary Hospital, 34-500 Zakopane, Poland; 3 Department of Pathology, Pulmonary Hospital, 34-500 Zakopane, Poland; 4 Bronchoscopy Unit, Pulmonary Hospital, 34-500 Zakopane, Poland; 5 Department of Pediatric Surgery, Institute of Pediatrics, Jagiellonian University Medical College, 31-531 Krakow, Poland; 6 Radiotherapy Unit, Katowice Center of Oncology, 31-531 Krakow, Poland

**Keywords:** non-small cell lung cancer, NSCLC, staging, EBUS, EUS, TEMLA

## Abstract

**Objectives**: The aim of the study was to compare the diagnostic efficiency between combined endobronchial ultrasound (EBUS)/endoesophageal ultrasound (EUS) and transcervical extended mediastinal lymphadenectomy (TEMLA) for preoperative staging of mediastinal lymph nodes in non-small cell lung cancer (NSCLC). **Material and Methods**: Between June 2011 and December 2017, a single-institution prospective randomized trial was conducted, and 250 patients with cytologically confirmed NSCLC, clinical stages cI–IIIA, were included. Positron emission tomography/computed tomography (PET/CT) was performed in all patients. After exclusions, 204 patients were randomized into the EBUS/EUS or TEMLA arms. Patients without N2/N3 metastases after mediastinal staging underwent surgery. The diagnostic yield and complication rates of the EBUS/EUS and TEMLA groups were compared. **Results**: There were 103 patients in the EBUS/EUS group, and N2 metastases were found in nine cases (8.7%). Ninety-four patients underwent surgery; in six cases, previously unsuspected N2 metastases were revealed. One hundred and one patients were randomized to the TEMLA group, which detected N2/N3 metastases in 15 cases (15.1%). Three patients were not operated on due to postoperative complications following TEMLA. Eighty-three patients underwent surgery, and a single N2 metastatic nodule was detected in one case. The diagnostic sensitivity, specificity, accuracy, PPV, and NPV were 94%, 100%, 99%, 100%, and 99% for TEMLA, respectively, and 60%, 100%, 94%, 100%, and 94% for EBUS/EUS, respectively. There was a significant difference in sensitivity (60% vs. 94%) between the EBUS/EUS and TEMLA groups in favor of the TEMLA group. Postoperative complications occurred in 6/101 (6%) patients after TEMLA, while no complications were observed in the EBUS/EUS group. **Conclusions**: TEMLA demonstrated superior sensitivity for detecting N2/3 disease compared to EBUS/EUS in terms of diagnostic performance for mediastinal staging of cI–IIIA NSCLC. Due to its more invasive nature, TEMLA was associated with a higher number of complications compared with EBUS/EUS.

## Simple Summary

Lung cancer is the leading cause of cancer-related deaths worldwide, with poor survival largely due to late diagnosis and early metastasis. Non-small cell lung cancer (NSCLC) accounts for about 85% of cases, while small cell lung cancer (SCLC) makes up 15%. Initial spread typically involves the thoracic lymph nodes. Treatment depends on cancer stage, ranging from surgery in early stages to systemic therapy in advanced disease. Imaging (CT, PET-CT) is important but often insufficient, so invasive methods like EBUS-TBNA, EUS-FNA, and TEMLA are used for staging. This clinical trial compares the diagnostic accuracy of EBUS-TBNA + EUS-b-FNA versus TEMLA in NSCLC, aiming to tailor diagnostic strategies to individual patients and improve oncological outcomes.

## 1. Introduction

Lung cancer remains the leading cause of cancer-related mortality worldwide, with approximately 1.76 million deaths recorded in 2018. It is currently the second most commonly diagnosed malignancy in both men and women. The highest incidence rates are observed in Europe, North America, and East Asia, with a five-year survival rate in Europe averaging only 13%. Key factors contributing to poor treatment outcomes include late diagnosis and rapid tumor progression, often accompanied by metastasis [1,2].

Lung cancer is classified into two main types: non-small cell lung cancer (NSCLC), which accounts for approximately 85% of cases, and small cell lung cancer (SCLC), comprising the remaining 15%. According to the WHO classification (2015), malignant epithelial lung tumors include adenocarcinoma, small cell carcinoma, squamous cell carcinoma, and other subtypes [3]. Metastases in lung cancer most commonly initially appear in lymph nodes within the thoracic cavity. For NSCLC, radical surgical treatment involves resection of the tumor along with the affected lobe or entire lung. Metastases in lymph nodes within the lung (stage IIB–IIIA) do not preclude surgery, while metastases in mediastinal lymph nodes on the same side as the tumor (IIIA–IIIB) necessitate neoadjuvant chemotherapy or chemoradiotherapy, with surgery being an option if remission is achieved. Metastases in contralateral mediastinal lymph nodes or supraclavicular lymph nodes (N3, stage IIIB–IIIC) contraindicate surgical treatment and require systemic therapy involving chemotherapy and radiotherapy. In stage IV, with distant metastases, systemic treatment, including chemotherapy or other systemic oncologic methods, is employed [4,5,6,7,8,9].

Preoperative diagnostics rely heavily on imaging techniques such as computed tomography (CT) and positron emission tomography (PET-CT), although their efficacy in comprehensive staging is limited. Therefore, invasive endoscopic methods, such as EBUS-TBNA (endobronchial ultrasound–transbronchial needle aspiration), EUS-FNA (endoesophageal ultrasound–fine needle aspiration), or combined EBUS–EUS, are recommended [10,11]. Surgical methods include mediastinoscopy, VAMLA (video-assisted mediastinal lymphadenectomy), TEMLA (transcervical extended mediastinal lymphadenectomy), and video-assisted thoracoscopy [12,13]. Endosonographic and surgical methods for preoperative staging of NSCLC continue to improve, although limited prospective studies are available comparing their diagnostic efficacy [14,15,16]. This prospective, randomized clinical trial investigates the diagnostic performance of two approaches for assessing mediastinal lymph node metastases in patients with non-small cell lung cancer (NSCLC): combined EBUS-TBNA with EUS-b-FNA (endoesophageal ultrasound–fine needle aspiration using a single bronchoscope) and TEMLA.

## 2. Materials and Methods

### 2.1. Ethical Considerations

All participants received detailed information about the study and signed informed consent forms. Patients were informed about the diagnostic methods for lymph node evaluation and subsequent treatment based on histopathological results. The study protocol was approved by the Bioethics Committee of the Oncology Center in Gliwice (KB/430-44/14) and registered with ClinicalTrials.gov (NCT03188562). No ethical concerns were identified, as all diagnostic techniques were considered routine clinical practices.

### 2.2. Study Design

This is a prospective, randomized clinical trial. The study was conducted between June 2011 and December 2017. Study candidates included patients from the Department of Thoracic Surgery at the Pulmonary Hospital “Odrodzenie” in Zakopane, diagnosed with cytologically confirmed NSCLC (clinical stages cI-IIIA) and preliminarily qualified for surgery. Patients with NSCLC (clinical stages cI-IIIA) who consented to participate were referred for PET-CT [17]. Those with findings of distant metastases, other malignancies, or advanced NSCLC (beyond stage IIIA) or who had withdrawn consent were excluded.

**Figure 1:**
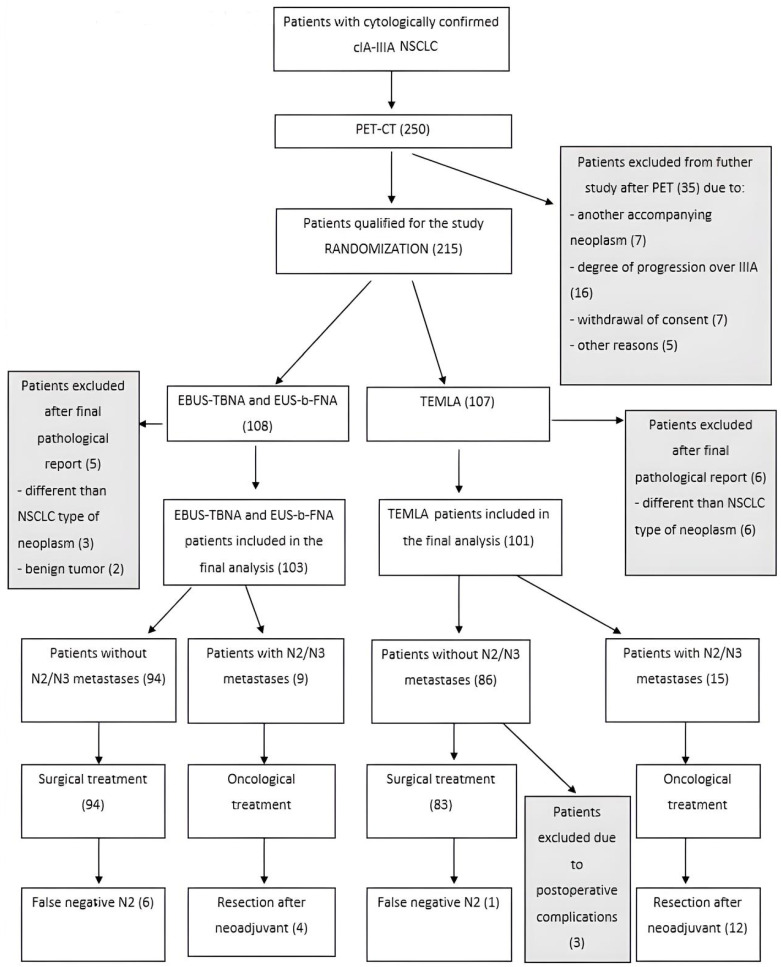
Algorithm of the trial.

The inclusion criteria for the study were cytologically or histologically confirmed NSCLC at clinical stages IA-IIIA; no severe comorbidities precluding surgery; and adequate respiratory function with FEV1 > 1.5 L for lobectomy and FEV1 > 2.0 L for pneumonectomy. The exclusion criteria were refusal to consent to treatment; NSCLC at clinical stages IIIB-IV; extensive mediastinal lymph node metastases on chest CT; insufficient cardiopulmonary function (NYHA class III-IV), presence of a second malignancy; and history of malignancy, except for non-melanoma skin cancer. Participants were randomized into two groups: the EBUS/EUS group and the TEMLA group. Patients with mediastinal lymph node metastases were treated with neoadjuvant chemotherapy (for N2 metastases) or definitive chemoradiotherapy (for N3 metastases). Patients without metastases underwent radical surgery (lobectomy or pneumonectomy with lymphadenectomy) using thoracotomy or video-assisted thoracic surgery (VATS) [8]. Diagnostic methods were validated with histopathological analysis of surgical specimens. The study flowchart is presented in Figure 1.

### 2.3. Sample Size Calculation

The sample size was determined based on the study’s primary aim—comparing the sensitivity of TEMLA and EBUS/EUS. Assuming a 5% type I error (two-sided) and 80% power, with prior sensitivity estimates of 95% for TEMLA and 80% for EBUS/EUS, a sample size of ~100 patients per group was calculated.

### 2.4. EBUS/EUS Techniques

**Figure 2:**
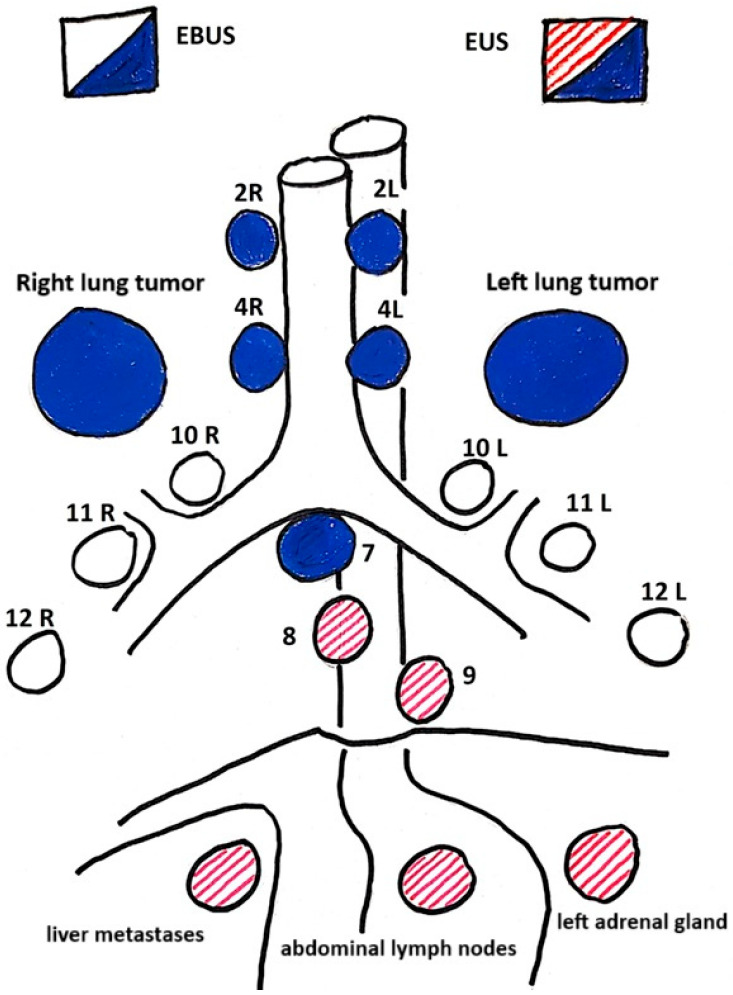
The complementary role of endobronchial ultrasound (EBUS) and endoscopic ultrasound (EUS) in the assessment of mediastinal lymph node staging.

EBUS and EUS are minimally invasive procedures for mediastinal lymph node staging in lung cancer. EBUS-TBNA enables access to lymph nodes near the trachea and bronchi, while EUS-B-FNA allows sampling of nodes near the esophagus and subdiaphragmatic areas. These techniques, used together, enable comprehensive mediastinal lymph node assessment, enhancing staging accuracy for lymph node metastases (N), primary tumors (T), and distant metastases (M) (Figure 2).

### 2.5. TEMLA Procedure

The TEMLA (transcervical extended mediastinal lymphadenectomy) procedure is performed under general anesthesia by experienced thoracic surgeons. A cervical incision (5–8 cm) allows access to the trachea following division of cervical muscles. A sternal lifting system is used, and a video mediastinoscope facilitates lymph node dissection along the trachea to the carina. Nodes from stations 7, 8, 2R, 4R, and 10R are removed en bloc, and the nodes of stations 4L, 2L, 6, and 5 are removed individually. The incision is closed in layers without mediastinal drains [18]. TEMLA integrates open surgery, video mediastinoscopy, and video thoracoscopy to achieve comprehensive lymphadenectomy. In some cases, TEMLA is combined with pulmonary resection in a single-stage or staged approach based on patient risk.

### 2.6. Pulmonary Resections

Anatomic pulmonary resections, including lobectomy, bilobectomy, and pneumonectomy, are performed via thoracotomy or minimally invasive VATS. Thoracotomy involves rib spreading, while VATS uses a 4–7 cm utility incision and ports for thoracoscopic instruments, with one or two additional small (1–2 cm) incisions, according to the surgeon’s preference. After anatomical identification, vessels and bronchi are ligated and divided with staples or sutures. Complete lymphadenectomy follows, targeting stations 2R, 4R, 7, 8, 9, 10R, and 11R on the right and 5, 6, 7, 8, 9, 10L, and 11L on the left [19]. Postoperatively, one or two chest drains are placed for fluid or air evacuation, typically removed between the third and fifth postoperative day.

### 2.7. Cytological and Histopathological Analyses

Specimens were analyzed in the Pathology Laboratory of the Pulmonary Hospital “Odrodzenie” in Zakopane. Cytology smears were fixed in alcohol and stained with hematoxylin and eosin (HE) for microscopic examination. For unclear cases, immunocytochemical tests were performed. Cytoblocks were prepared in the same manner as histological specimens, enabling precise immunohistochemical (IHC) and molecular diagnostics [17].

### 2.8. Postoperative Histopathological Processing

Tissue specimens were fixed in 10% neutral-buffered formalin for 24–72 h. Cytoblocks were processed for staging and predictive marker analysis, with thin sections stained with HE. Larger samples were divided for comprehensive analysis, ensuring adequacy for both diagnosis and predictive testing. Standardized processing enhances diagnostic accuracy and supports targeted therapy selection.

## 3. Results

### 3.1. Study Framework

The study, conducted between June 2011 and December 2017, included 250 patients with confirmed NSCLC (clinical stages cI-IIIA) who underwent PET-CT. After exclusions, 215 patients were randomized into the TEMLA (*n* = 107) and EBUS/EUS (*n* = 108) groups. Following the final histopathological assessment, only patients with confirmed NSCLC were included in the final analysis, resulting in 204 patients: TEMLA (*n* = 101) and EBUS/EUS (*n* = 103) (Figure 1). No significant differences regarding age and gender between groups were found.

### 3.2. EBUS/EUS Group

Lymph node stations were assessed following guidelines, with suspicious nodes biopsied using EBUS-TBNA or EUS-B-FNA. N2 metastases were confirmed in 8.7% (9/103), and these patients received neoadjuvant chemotherapy. No N3 metastases were detected. Anatomical lung resections with lymphadenectomy were performed in 94/94 eligible patients. Metastases were found in 6.4% (6/94), including stations beyond the scope of EBUS/EUS (twice in stations 7 and 4R, and once in stations 5 and 5 + 6).

### 3.3. TEMLA Group

Extensive mediastinal lymphadenectomy was performed, retrieving an average of 27.3 lymph nodes per procedure. N2/N3 metastases were detected in 15.1% (15/101), and these patients received neoadjuvant therapy. Complications (e.g., pulmonary embolism, reduced spirometric values) prevented surgery in 3.5% (3/86). Radical pulmonary resection was performed in 96.5% (83/86) of cases, with a metastasis rate of 1.2% (1/83), including a case in a station beyond the scope of TEMLA (station 9).

### 3.4. Comparative Outcomes

Patients with N2 metastases received neoadjuvant chemotherapy, with subsequent radical pulmonary resection performed in 44% (4/9) of EBUS/EUS patients and 80% (12/15) of TEMLA patients. Overall, the numbers of lung resections were comparable: EBUS/EUS (95.1%) vs. TEMLA (94.1%) (*p* = 0.75).

### 3.5. Histopathological Tumor Types Analysis

Adenocarcinoma was the most frequent tumor type in the cohort (51%), followed by squamous cell carcinoma (44%). Other tumor types, including large cell carcinoma, large-cell neuroendocrine carcinoma, adenosquamous carcinoma, squamous-neuroendocrine carcinoma, and NSCLC NOS (not otherwise specified), collectively accounted for 4.4% of cases. The distribution of histopathological types did not significantly differ between the EBUS/EUS and TEMLA groups (Pearson’s χ^2^ = 0.28, *p* = 0.87), even when stratified by gender.

### 3.6. Tumor Localization Analysis

The most common tumor locations were the right upper lobe (31.4%) and left upper lobe (25.5%), followed by the right lower (16.2%) and left lower lobes (15.7%). Twenty patients had extensive tumors categorized as “whole lung tumors” (12 in the EBUS/EUS group, 8 in TEMLA). Three patients (1.5%) had tumors in the right middle lobe. No significant differences in tumor location frequencies were found between the EBUS/EUS and TEMLA groups (Pearson’s χ^2^ = 3.5, *p* = 0.62). ANOVA showed no significant association between tumor location and patient age (F(4, 196) = 0.61, *p* = 0.65).

### 3.7. Clinical Stage Analysis

**Figure 3:**
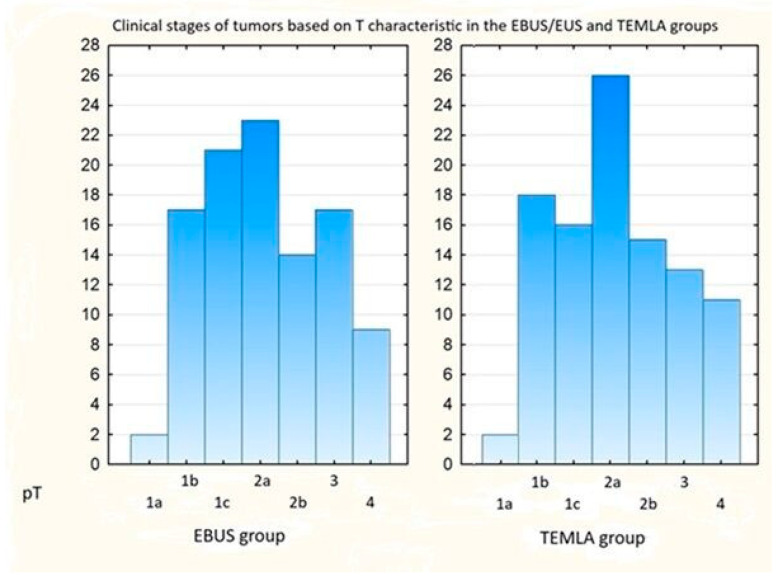
Clinical stages of tumors based on T characteristics in the EBUS/EUS and TEMLA groups.

The clinical stage was analyzed in 103 patients from the EBUS/EUS group and 101 from the TEMLA group. No significant differences in clinical stage were observed between the groups (Pearson’s χ^2^ = 2.97, *p* = 0.89) (Table 1, Figure 3).

### 3.8. Analysis of Diagnostic Methods

#### 3.8.1. EBUS/EUS Group

In the EBUS/EUS group, 94 patients without detected lymph node metastases after endobronchial (EBUS) and esophageal (EUS) ultrasound underwent anatomical lung resection with systematic lymphadenectomy. Nine patients with detected metastases received neoadjuvant chemotherapy. Postoperative histopathological analysis of resected lymph nodes served as the reference standard (“gold standard”) to evaluate PET-CT, CT, and EBUS/EUS diagnostic performance.

**Figure 4:**
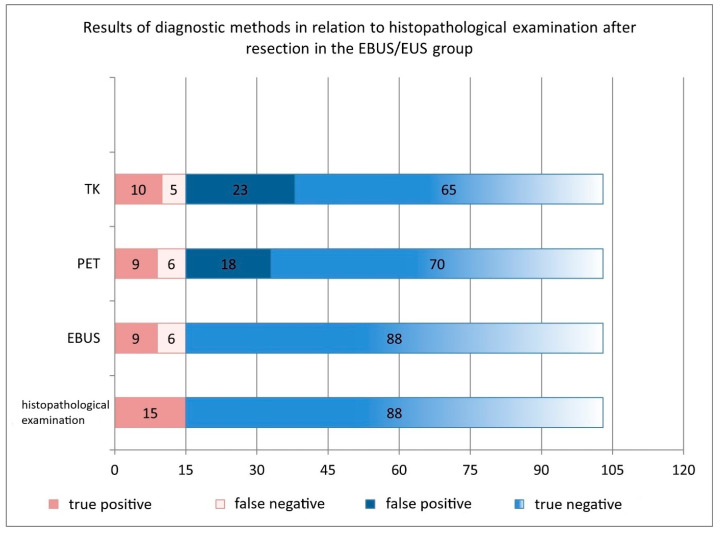
Comparison of the diagnostic performance of PET-CT, CT, and EBUS/EUS in mediastinal lymph node staging of NSCLC in the EBUS/EUS group.

The sensitivity, specificity, accuracy, PPV and NPV for EBUS/EUS were 60%, 100%, 94%, 100%, and 94%, respectively. PET-CT had a sensitivity, specificity, accuracy, PPV, and NPV of 60%, 80%, 77%, 33%, and 92%, respectively, while CT results were 67%, 74%, 64%, 30%, and 93%, respectively. EBUS/EUS demonstrated significantly higher specificity, accuracy, and PPV compared to PET-CT and CT. Metastases were identified in 15% of patients, while PET-CT and CT showed false positives (18% and 22%) and false negatives (6% and 5%) (Table 2, Figure 4).

#### 3.8.2. TEMLA Group

**Figure 5:**
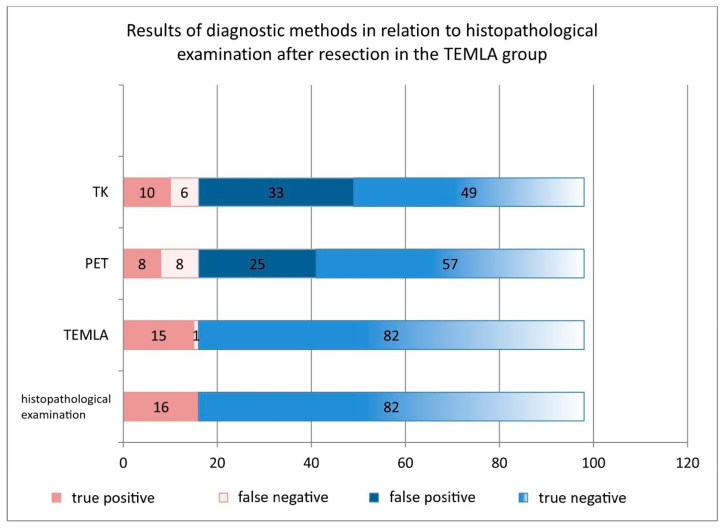
Comparison of the diagnostic performance of PET-CT, CT, and TEMLA in assessing the mediastinal lymph node staging of NSCLC in the TEMLA group.

In the TEMLA group, 86 patients with no detected metastases underwent lung resection with lymphadenectomy, while 15 patients with confirmed metastases received oncologic treatment. TEMLA had a sensitivity, specificity, accuracy, PPV, and NPV of 94%, 100%, 99%, 100%, and 99%, respectively, outperforming PET-CT and CT. PET-CT results included false positives (FP) in 25% and false negatives (FN) in 8%, with an overall accuracy of 66%. CT had FP in 34% and FN in 6%, with an accuracy of 60% (Table 3, Figure 5).

### 3.9. Comparative Analysis of TEMLA and EBUS/EUS

**Figure 6:**
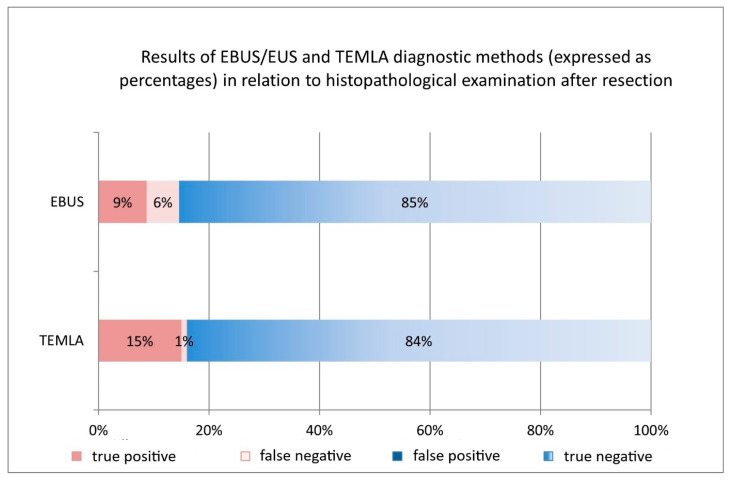
Comparison of the diagnostic performance of EBUS/EUS and TEMLA in assessing the mediastinal lymph node staging of NSCLC.

Both TEMLA and EBUS/EUS are effective for assessing mediastinal lymph node involvement in NSCLC. Both methods achieved 100% specificity and PPV. TEMLA had significantly higher sensitivity (94%) than EBUS/EUS (60%) (*p* = 0.0234). Accuracy and NPV were similar, with no statistically significant differences (*p* > 0.05) (Table 4, Figure 6).

### 3.10. Complications After Staging

In the EBUS/EUS group, patients experienced typical post-procedural symptoms such as sore throat, hoarseness, transient low-grade fever, and occasional mild hemoptysis following the biopsy. No major complications were observed in the postoperative period.

In the TEMLA group, complications were observed in 6% of patients (6/101): two cases of transient recurrent laryngeal nerve palsy (2%), two cases of decreased postoperative spirometry values (2%), one case of bleeding requiring re-mediastinoscopy (1%), and one case of pulmonary embolism (1%). In this group, 3.5% of patients (3/86) were not qualified for lung resection surgery, including two patients with reduced spirometry values and one patient who experienced a pulmonary embolism.

### 3.11. Complications After Pulmonary Resections

There were no significant differences in postoperative complication rates between the groups (Table 5). In the EBUS/EUS group, according to the Clavien–Dindo classification, there were three Grade II complications, five Grade III, three Grade IV, and no Grade V complications. In the TEMLA group, there were four Grade II, four Grade III, three Grade IV, and one Grade V complication (Table 6).

Definitions (Clavien–Dindo):

- Grade II—Requires pharmacological treatment (e.g., antibiotics, blood transfusions).
- Grade III—Requires surgical, endoscopic or radiological intervention.
- Grade IV—Life-threatening complications requiring ICU management.
- Grade V—Death of a patient.

## 4. Discussion

The primary aim of this study was to compare the diagnostic performance of the surgical TEMLA method and endosonographic techniques (EBUS/EUS) for staging mediastinal lymph nodes in NSCLC patients. TEMLA demonstrated a higher sensitivity (94%) compared to EBUS/EUS (60%, *p* = 0.0234), while both methods showed similar specificity, accuracy, PPV, and NPV. TEMLA offers the advantage of more precise detection of mediastinal metastases and allows for extended lymphadenectomy, which can provide therapeutic benefits. However, its invasive nature limits its routine use compared to EBUS/EUS, which is less invasive and more accessible. The study highlighted the limitations of imaging methods such as CT and PET-CT, which exhibited high rates of FN and FP in the absence of tissue sampling [20,21,22]. According to other authors, combining endosonographic and surgical methods was shown to improve diagnostic accuracy. For instance, adding EUS-FNA to mediastinoscopy increased the detection of mediastinal metastases by 12% and reduced unnecessary thoracotomies by 16% [23]. TEMLA surpassed mediastinoscopy in diagnostic efficacy by enabling systematic lymph node removal, which reduces FN rates and improves staging accuracy compared to biopsies performed during mediastinoscopy [24].

Despite advancements in non-invasive imaging and endosonographic techniques, invasive procedures like TEMLA remain essential for accurate staging, particularly in advanced NSCLC cases. Current guidelines recommend EBUS/EUS as the first-line diagnostic method, reserving surgical staging for cases requiring further confirmation. Over the last two decades, the integration of imaging, endosonography, and surgical techniques has significantly improved mediastinal staging. The 2014 ESTS guidelines highlight the importance of combining endosonographic and advanced surgical methods like TEMLA to achieve optimal diagnostic accuracy.

TEMLA offers superior diagnostic performance for staging NSCLC compared to EBUS/EUS and mediastinoscopy, particularly in detecting mediastinal lymph node metastases [25]. While endosonographic techniques remain the preferred first-line option due to their minimally invasive nature, TEMLA is essential for cases requiring comprehensive mediastinal evaluation or therapeutic lymphadenectomy.

According to the ninth edition of the TNM classification for lung cancer, both endoscopic techniques (EBUS/EUS) and surgical approaches (such as TEMLA) enable accurate assessment of mediastinal nodal involvement, including differentiation between N2a and N2b stages. In the present study, the EBUS/EUS group demonstrated four cases of N2a and five cases of N2b, while the TEMLA group identified four cases of N2a and 11 cases of N2b. The higher detection rate of N2b in the TEMLA group is attributable to the greater number of lymph nodes resected during the procedure (mean of 27.3 nodes) compared to the number of nodes sampled during EBUS/EUS.

A follow-up to the current study, planned as the next step in the investigation, will analyze five-year survival rates in the examined patient cohort and assess the potential impact of TEMLA on five-year survival in NSCLC patients compared to the EBUS/EUS group. In the EBUS/EUS group, 59% (61/103) of patients underwent lymph node verification using EBUS-TBNA or EUS-b-FNA, while 41% (42/103) of procedures were completed without biopsy. No complications were observed in either group, consistent with literature reports indicating a complication rate below 1%. A 2020 study by Kayawake et al. reported a 0.97% complication rate after EBUS-TBNA, including mediastinitis, severe pneumonia, and bronchial obstruction. In the present study, no mortality was noted following EBUS/EUS [26].

The TEMLA, a more invasive procedure requiring general anesthesia and extensive mediastinal exploration, showed a 6% complication rate (6/101). TEMLA complications were higher than those reported for mediastinoscopy (0.6–1.07%) but involved no life-threatening vascular injuries due to better visualization. Only 3.5% (3/86) of TEMLA patients experienced clinical deterioration preventing subsequent lung resection, and 96.5% (83/86) proceeded to surgery, a rate comparable to mediastinoscopy (91.3–91.9%).

There is one important remark that should be added. Despite the lower complication rate for the EBUS/EUS technique in comparison to the TEMLA group, the possibility of biopsy of stations 5 and 6 is very limited for EBUS/EUS. In a study where such attempts were made, there was a very serious complication reported—a delayed aortic pseudoaneurysm, which showed the risk of such endoscopic biopsy. There has been no such life-threatening complication encountered in the group of 928 patients who underwent TEMLA for staging of NSCLC [27].

The average duration of TEMLA was 94.5 min (range 55–210), longer than VAMLA (54 min) and re-mediastinoscopy (53 min) due to its comprehensive lymphadenectomy. No mortality was observed after TEMLA in the current study, aligning with previous reports showing a 0.4% mortality rate (for medical reasons only) [27].

Postoperative mortality following lung resection and lymphadenectomy was 0.6% (1/177), involving a myocardial infarction after left lower lobectomy in a patient staged with TEMLA. This is consistent with reported surgical mortality rates of 1–3% for lobectomy and ≥5% for pneumonectomy. Overall postoperative complications occurred in 12% (22/177) of patients, with similar rates in the EBUS (12%) and TEMLA (13%) groups. No significant difference was observed between the groups.

Thoracic surgery patients are typically high-risk due to advanced age, comorbidities, poor physical condition, malnutrition, and smoking history, often presenting with impaired pulmonary function. These factors, combined with the invasive nature of surgery, prolonged anesthesia, and intensive care requirements, significantly increase perioperative risks. The mortality and complication rates observed in this study align with published data.

The single-center study design somewhat limits the generalizability of the findings to a broader patient population. However, it is important to note that the analyzed groups were homogeneous in terms of the distribution of histopathological types, tumor location frequencies, and clinical stage and reflected the statistical distribution of NSCLC in the general population, which should not pose a barrier to the potential application of study results in clinical practice.

Currently, the main indication for TEMLA is mediastinal staging in patients with locally advanced but potentially resectable lung cancer and negative EBUS/EUS findings, regardless of chest CT or PET/CT results [3,4,5]. A second indication is restaging after neoadjuvant therapy in NSCLC with negative EBUS/EUS [6]. A third is the presence of a resectable tumor with suspected N1/N2 involvement despite negative EBUS/EUS. In such cases, TEMLA may be combined with intraoperative lymph node assessment and VATS lobectomy. TEMLA is also preferred when stations 5 and 6 are suspicious, as these are nearly inaccessible via EBUS/EUS.

## 5. Conclusions

The reliability of imaging studies (CT and PET/CT) is limited for diagnosing mediastinal metastases in lung cancer, necessitating invasive diagnostic methods with pathological sampling. TEMLA demonstrated significantly higher diagnostic sensitivity compared to combined EBUS/EUS for detecting NSCLC metastases in mediastinal lymph nodes. The complication rate of TEMLA is higher than that of EBUS/EUS.

EBUS/EUS should be the first-line staging technique due to its less invasive nature compared to mediastinoscopy or TEMLA. However, in cases of suspicious lymph nodes, TEMLA should be considered as a confirmatory procedure to prevent unnecessary surgical treatment.

## Figures and Tables

**Table 1:** Pathological stage based on T characteristics in the EBUS and TEMLA groups.

	T1a	T1b	T1c	T2a	T2b	T3	T4	Total
**EBUS/EUS**	2 (1.9%)	17 (16.5%)	21 (20.4%)	23 (22.3%)	14 (13.6%)	17 (16.5%)	9 (8.7%)	103
**TEMLA**	2 (2%)	18 (17.8%)	16 (15.8%)	26 (25.7%)	15 (14.8%)	13 (12.8%)	11 (10.9%)	101
**Overall**	4	35	37	49	29	30	20	204

**Table 2:** Comparison of the diagnostic performance of PET-CT, CT, and EBUS/EUS in assessing the mediastinal lymph node staging of NSCLC in the EBUS/EUS group.

	Sensitivity (95% CI)	Specificity (95% CI)	Accuracy (95% CI)	Positive Predictive Value (95% CI)	Negative Predictive Value (95% CI)
**EBUS/EUS**	**60%** (32–84%)	**100%** (95–100%)	**94%** (87–98%)	**100%** (66–100%)	**94%** (87–98%)
**PET**	**60%** (32–84%)	**80%** (69–87%)	**77%** (67–84%)	**33%** (16–53%)	**92%** (83–97%)
**TK**	**67%** (42–90%)	**74%** (63–82%)	**64%** (54–73%)	**30%** (13–50%)	**93%** (84–98%)

**Table 3:** Comparison of the diagnostic performance of PET-CT, CT, and TEMLA in assessing the mediastinal lymph node staging of NSCLC in the TEMLA group.

	Sensitivity (95% CI)	Specificity (95% CI)	Accuracy (95% CI)	Positive Predictive Value (95% CI)	Negative Predictive Value (95% CI)
**TEMLA**	**94%** (71–100%)	**100%** (96–100%)	**99%** (93–100%)	**100%** (78–100%)	**99%** (93–100%)
**PET-TK**	**50%** (25–75%)	**70%** (58–79%)	**66%** (56–75%)	**24%** (11–42%)	**88%** (77–94%)
**TK**	**63%** (35–85%)	**60%** (48–70%)	**60%** (48–70%)	**23%** (12–39%)	**89%** (78–96%)

**Table 4:** Comparison of diagnostic performance in the EBUS/EUS and TEMLA groups.

	Sensitivity (95% CI)	Specificity (95% CI)	Accuracy (95% CI)	Positive Predictive Value (95% CI)	Negative Predictive Value (95% CI)
**TEMLA**	**94%** (71–100%)	**100%** (96–100%)	**99%** (93–100%)	**100%** (78–100%)	**99%** (93–100%)
**EBUS/EUS**	**60%** (32–84%)	**100%** (95–100%)	**94%** (87–98%)	**100%** (66–100%)	**94%** (87–98%)

**Table 5:** Complications after lung resections in the EBUS/EUS and TEMLA groups.

Type of Complications	EBUS/EUS Group (94)	TEMLA Group (83)	Difference (*p*)
Postoperative bleeding	3	4	*p* = 0.6
Pneumonia	1	2	*p* = 0.5
Surgical wound infection	1	2	*p* = 0.5
Stroke	1	1	*p* = 0.9
Bronchopleural fistula after pneumonectomy	1	0	*p* = 0.3
Cardiac arrest	1	0	*p* = 0.3
Pulmonary embolism	1	0	*p* = 0.3
Myocardial infarction	0	1	*p* = 0.3
Phrenic nerve palsy	0	1	*p* = 0.3
Pulmonary edema	1	0	*p* = 0.3
Air leak requiring re-thoracotomy	1	0	*p* = 0.3
Mortality	0	1	*p* = 0.3

**Table 6:** Postoperative complications according to Clavien–Dindo classification.

Clavien–Dindo Grade	EBUS/EUS Group (*n* = 94)	TEMLA Group (*n* = 83)
Grade II	3	4
Grade III	5	4
Grade IV	3	3
Grade V	0	1
Total Complications	11 (11.7%)	12 (14.5%)

## Data Availability

The study protocol was registered at ClinicalTrials.gov (NCT03188562). https://ichgcp.net/clinical-trials-registry/NCT03188562#google_vignette, (accessed on 1 February 2014).

## Abbreviations

The following abbreviations are used in this manuscript:

ANOVA	One-way analysis of variance
EBUS	Endobronchial ultrasound
EBUS-TBNA	Endobronchial ultrasound-guided transbronchial needle aspiration
EUS	Endoesophageal ultrasound
EUS-FNA	Endoesophageal ultrasound—fine needle aspiration
EUS-b-FNA	Endoesophageal ultrasound—fine needle aspiration by use of single bronchoscope
FN	False negative result
FP	False positive result
MANOVA	Multivariate analysis of variance
NOS	Not otherwise specified NSCLC
NPV	Negative predictive value
NSCLC	Non-small cell lung cancer
PET-CT	Positron emission tomography/computed tomography
PPV	Positive predictive value
SCLC	Small cell lung cancer
TEMLA	Transcervical extended mediastinal lymphadenectomy
CT	Computed tomography
TN	True negative result
TP	True positive result
VAMLA	Video-assisted mediastinoscopic lymphadenectomy
VATS	Video-assisted thoracic surgery
